# Protocol for a systematic review and individual participant data meta-analysis of B-type natriuretic peptide-guided therapy for heart failure

**DOI:** 10.1186/2046-4053-3-41

**Published:** 2014-05-02

**Authors:** Maria Pufulete, Julian PT Higgins, Chris A Rogers, Lucy Dreyer, William Hollingworth, Mark Dayer, Angus Nightingale, Theresa McDonagh, Barnaby C Reeves

**Affiliations:** 1 Clinical Trials and Evaluation Unit, University of Bristol, Level 7, Bristol Royal Infirmary, Queen's Building, Bristol BS2 8HW, UK; 2 School of Social and Community Medicine, University of Bristol, Canynge Hall, 39 Whatley Road, Bristol BS8 2PS, UK; 3 Department of Cardiology, Taunton and Somerset NHS Foundation Trust, Musgrove Park, Taunton, Somerset TN2 6A, UK; 4 Department of Cardiology, Bristol Heart Institute, Bristol Royal Infirmary, Bristol BS2 8HW, UK; 5 Cardiovascular Division, King’s College London, King’s College Hospital, Denmark Hill, London SE5 9RS, UK

**Keywords:** Heart failure, B-type natriuretic peptide, individual participant data meta-analysis

## Abstract

**Background:**

Several aggregate data meta-analyses suggest that treatment guided by the serum concentration of natriuretic peptides (B-type natriuretic peptide (BNP) or its derivative N-terminal pro-B-type natriuretic peptide (NT-BNP)) reduces all-cause mortality compared with usual care in patients with heart failure (HF). We propose to conduct a meta-analysis using individual participant data (IPD) to estimate the effect of BNP-guided therapy on clinical outcomes, and estimate the extent of effect modification for clinically important subgroups.

**Methods:**

We will use standard systematic review methods to identify relevant trials and assess study quality. We will include all randomized controlled trials (RCTs) of BNP-guided treatment for HF that report a clinical outcome. The primary outcome will be time to all-cause mortality. We will collate anonymized, individual patient data into a single database, and carry out appropriate data checks. We will use fixed-effects and random-effects meta-analysis methods to combine hazard ratios (HR) estimated within each RCT, across all RCTs. We will also include a meta-analysis and meta-regression analyses based on aggregate data, and combine IPD with aggregate data if we obtain IPD for a subset of trials.

**Discussion:**

The IPD meta-analysis will allow us to estimate how patient characteristics modify treatment benefit, and to identify relevant subgroups of patients who are likely to benefit most from BNP-guided therapy. This is important because aggregate meta-analyses have suggested that clinically relevant subgroup effects exist, but these analyses have been unable to quantify the effects reliably or precisely.

**Trials registration:**

PROSPERO 2013: CRD42013005335

## Background

Heart failure (HF) is one of the most costly conditions to manage, and markedly impairs the patient's quality of life. It has an estimated prevalence of 6 to 10% in people over 65 years of age [1], increasing to 14% in people over 85 years [2]. Prevalence is expected to increase in the future because of the ageing population and improved survival of people with ischemic heart disease. The prognosis of patients with HF is poor; up to 40% of newly diagnosed patients die within 1 year [3,4].

Pharmacological treatment for HF is complex, and includes angiotensin-converting enzyme (ACE) inhibitors, angiotensin receptor blockers, beta-blockers, and mineralocorticoid receptor antagonists. Clinical guidelines recommend up-titration of these drugs to target (or maximally tolerated) doses, but this is difficult to achieve in practice, given the number of drugs involved and the fact that the sequence of addition and up-titration is based largely on clinician judgement. The use of biomarker test results to guide up-titration of medication has been proposed as a more objective means of achieving optimal therapy in patients with HF.

Several randomized controlled trials (RCTs) have assessed whether using serum natriuretic peptide levels (B-type natriuretic peptide (BNP) or its derivative N-terminal pro-B-type natriuretic peptide (NT-BNP); collectively referred to here as 'BNP') to guide up-titration of medication improves clinical outcomes. BNP reflects cardiac function. BNP levels rise in line with the severity of symptoms [5] and are lowered by drugs used to treat HF, and this sequence (monitoring and consequent changes in medication) is associated with improved clinical outcome [6-9]. BNP predicts adverse outcomes in HF and provides powerful risk stratification with respect to mortality across the entire range of HF stages [10].

To date there have been four aggregate data meta-analyses of RCTs of BNP-guided therapy, which assessed 6 (n = 1627), 8 (n = 1726), 11 (n = 2414) and 12 (n = 2686) RCTs [6-9], respectively, with patients followed for an average of 12 to 16 months. All reported a reduction in all-cause mortality in the BNP-guided therapy group compared with usual care (symptom-based therapy) (hazard ratio (HR) = 0.69, 95% confidence interval (CI) 0.55 to 0.86 [6]; risk ratio (RR) = 0.76, 95% CI 0.63 to 0.91 [7]; RR = 0.83, 95% CI 0.69 to 0.99 [8]; odds ratio (OR) = 0.74, 95% CI 0.60 to 0.91 [9]). The two latter meta-analyses, which assessed 11 and 12 RCTs, respectively, also reported a decrease in HF-related rehospitalization (RR = 0.65, 95% CI 0.50 to 0.84 [8]; OR = 0.55, 95% CI 0.40 to 0.77 [9]). In subgroup analyses of three meta-analyses [7-9] the reduction in mortality was observed only in younger patients (≤75 years), although none of these meta-analyses formally quantified treatment effect modification. The identification of relevant treatment subgroups is important because it is known that some patients do not benefit from, and may even be harmed by, intensive drug therapy for HF. For example, in elderly patients with multiple comorbidities, the risks of adverse outcomes from intensified therapy might outweigh any benefits, because up-titration of diuretics, ACE inhibitors, and beta-blockers may worsen clinical outcomes in such patients by causing hypotension and aggravating renal failure [11].

The main limitation of aggregate data meta-analysis is that variation in treatment effects across individuals with different effect modifiers cannot be explored. If subgroup analyses are presented, the definition of the subgroups may vary across trials, and results may be reported inconsistently, or be incompatible in other ways across trials (for example, outcomes reported at different points in time or with different periods of follow-up). Findings may also be selectively reported, so that subgroup effects are presented only when they are statistically significant. Individual participant data (IPD) meta-analysis uses ‘raw’ individual-level data obtained from each study to estimate an overall effect, avoiding the limitations arising from inconsistencies in conduct and reporting across trials. In particular, a powerful and detailed analysis of treatment effect modification can be undertaken, using within-trial data to estimate how the characteristics of the patients modify treatment benefit [12].

IPD meta-analysis is widely regarded as the gold standard, and has several other advantages over aggregate data meta-analysis. It allows a more flexible analysis of outcomes, including time-to-event and subgroup analyses, detailed data checking to ensure consistency, and an assessment of the quality of randomization and follow-up. The IPD can also be updated with more recent follow-up information. We propose to conduct an IPD meta-analysis to determine whether BNP-guided therapy improves outcomes in patients with HF. This will also allow us to determine which subgroups of patients are likely to benefit most from BNP-guided therapy, and possibly identify specific components of the interventions responsible for the improved outcomes observed in individual RCTs. An existing IPD meta-analysis of 8 RCTs (including 2,000 patients) was published recently [13]. We will produce an up-to-date IPD meta-analysis using data from more recent trials in accordance with this protocol. If we do not obtain IPD for all the eligible trials, we will combine IPD with aggregate data (for trials that have not provided IPD), so that we can still exploit the benefits of IPD while synthesizing evidence from all the available studies [14].

### Aims and objectives

The main aim of the IPD meta-analysis is to determine the clinical effectiveness of BNP-guided therapy versus standard care. Specific objectives are as follows:

1. To estimate the effect of treatment guided by serial BNP monitoring on clinical outcomes (time to all-cause mortality, death related to HF, cardiovascular death, all-cause hospital admission, hospital admission for HF, adverse events, quality of life) compared with usual care (symptom-based therapy).

2. To estimate the extent of effect modification for key outcomes including all-cause mortality and hospital admission for clinically important subgroups. The following subgroups have been specified through consensus with clinical members of the review team: age, gender, type of HF, severity of HF, baseline BNP levels, New York Heart Association (NYHA) class, left ventricular ejection fraction (LVEF), blood pressure (BP), body mass index (BMI), atrial fibrillation (AF), diabetes, cardiomyopathy, and risk score for mortality.

3. To quantify the extent to which improved outcomes are explained by up-titration of medication and/or reduction in BNP levels. This may be possible only for a subset of trials, or not at all, depending on the availability of IPD describing titration of medication in trial datasets.

4. To combine adverse event and discontinuation data to describe the safety of BNP-guided therapy in patients with HF.

## Methods

The IPD meta-analysis will be conducted in accordance with the methods recommended by the IPD Meta-analysis Methods Group of the Cochrane Collaboration [15] and other published guidelines [16].

### Criteria for considering studies for the review

#### Types of studies

We will include RCTs of BNP-guided treatment for HF that report an eligible outcome (see below).

#### Population

We will include all patients aged over 18 years who are being treated for HF in primary or secondary care.

#### Interventions

Treatment guided by serial BNP or NT-BNP measurements (BNP-guided therapy) or treatment guided by clinical assessment (usual care).

#### Outcomes

The primary outcome for the IPD meta-analysis will be time to all-cause mortality. Secondary outcomes are: death related to HF, cardiovascular death, all-cause hospital admission, hospital admission for HF, adverse events, and quality of life.

For studies that do not provide IPD, we will collect aggregate data relating to four levels of specification of outcome measures as proposed by Zarin *et al*. [17]: domain (for example quality of life), specific metric (for example Living with Heart Failure Minnesota Questionnaire), specific metric used to characterize each participant’s results (for example change from baseline), and method of aggregation (for example mean change and 95% CI). We have grouped the outcomes into the following domains: all-cause mortality, cause-specific mortality (for death related to HF and death from any cardiovascular cause); harms (all-cause hospital admission, hospital admission for HF, other serious adverse events); and quality of life (see Quality Assessment section for justification for these domains).

#### Language

No language restrictions will apply.

### Search methods for identification of studies

We initially used published systematic reviews [6-9] to identify relevant trials. We will search the following electronic databases: MEDLINE, EMBASE, The Cochrane Library and ISI Web of Science (Citations Index and Conference Proceedings) using the search strategy shown in Additional file 1. We will search the World Health Organization International Clinical Trials Registry Platform (WHO ICTRP; http://apps.who.int/trialsearch/) and Current Controlled Trials (http://www.controlled-trials.com) to identify trials in progress. We will also review the reference lists of all full-text papers and correspond with all trial authors to identify any trials that we may have missed.

### Inclusion of studies

#### Study selection

Two members of the review team will independently triage the titles and abstracts identified by the search to remove those that are clearly inappropriate. The remaining papers will have clear inclusion criteria applied to them. Disagreements about study inclusion will be resolved by discussion with a third review author. All trials excluded from the review will be given reasons for exclusion, such as 'not a randomized trial' or 'inappropriate control'. All RCTs in languages other than English will be translated into English. The English texts of RCTs reported in other languages will be made available to all collaborators.

### Establishing a collaboration

Authors of eligible studies will be invited to join the collaboration by providing us with IPD. We will identify contact information from the published RCT or an online search. We will contact the main trial author (corresponding author) and provide them with the IPD meta-analysis protocol and a cover letter explaining what the study is about. If we receive no response from the corresponding author, another investigator from the study will be contacted.

#### Quality assessment

We will assess studies as having a low, unclear, or high risk of bias within the following domains: random sequence generation (selection bias); allocation concealment (selection bias); blinding of participants, personnel, and outcome assessors (performance and detection bias); incomplete outcome data (attrition bias); selective outcome reporting (reporting bias); and other sources of bias. This assessment is based on current Cochrane Collaboration guidelines [18], but if any updated guidelines are published by the time of reporting, we will use those. For blinding and incomplete outcome data, we will assess risk of bias separately for the pre-specified outcome domains (all-cause mortality, cause-specific mortality, adverse events, and quality of life). We have separated all-cause mortality from cause-specific mortality because cause-specific mortality may be at risk of bias, depending on whether or not the person assigning the cause of death was blinded to the allocated intervention.

Based on study reports, we will collect preliminary information to inform the risk of bias assessments. Trial authors (collaborators) will be asked to provide a study protocol, if available. If not, they will be asked to clarify uncertainties arising from reporting of their study with respect to information needed for the risk of bias assessment.

Two members of the review team will independently assess the risk of bias in each included study from all available information using the domain-based evaluation tool described in the *Cochrane Handbook for Systematic Reviews of Interventions*[18]. Disagreements will be resolved by discussion with a third review author. The assessment for incomplete outcome data and selective outcome reporting will be used only for studies for which IPD is not provided. Blinding of participants, healthcare providers, and outcome assessors will be judged as low risk for mortality, which is an objective outcome.

We will search the grey literature extensively to check for publication bias. We will contact all authors of relevant unpublished studies and request data from their trials. Publication bias in studies included in the meta-analysis will be evaluated with a funnel plot (a plot of the effect sizes against their standard errors) and trend tests.

### Development of the database

#### Data collection

IPD will be sought from all included RCTs and collated into a single database. We will request the data for all randomized patients. We will provide authors with a list of data items we require (Table 1), but we will accept trial data in all formats in order to minimize the amount of work for trial authors and to ensure maximum participation. We will also request the formal data dictionary for the dataset (table of information about the data elements), the data collection schedule (time points at which data were collected) and 'process' data describing actions conditional on BNP measurements.

**Table 1:** List of data items required

**Type of data**	
**Study level**	Country in which study was carried out
	Number of participants randomized
	Number allocated to the BNP group
	Number allocated to the standard care group
	Setting (primary care, hospitals, specialist clinics)
	Date first patient randomized
	Date final patient randomized
	Date of final follow-up
	Did the study measure quality of life (state tool that was used)
	Did the study measure heart failure risk score (state tool that was used)
	Details of intervention (frequency of testing, actions, etc.)
	Details of comparator (frequency of review, actions, etc.)
**Individual participant**	**Variables collected at study entry**
*Demography*	Age
	Sex
	Body mass index (or weight and height)
	Smoking status
	Date of entry into study/date of randomization
	Allocated to BNP or standard care
	Centre if multi centre
*Medical history*	Cause of heart failure
	Previous myocardial infarction
	Previous intervention (PCI/CABG)
	Previous stroke
	Previous angina pectoris
	Previous peripheral artery disease
	Diabetes status (including type)
	History of hypertension
	History of atrial fibrillation
	History of chronic obstructive pulmonary disease
	Pacemaker *in situ*
	Implantable cardioverter defibrillator *in situ*
	Heart failure risk score (if measured)
**Individual participant**	**Variables collected at each visit (data required for all visits including baseline visit)**
	Date of visit
*Clinical*	NYHA class
	Left ventricular ejection fraction
	Resting heart rate
	Systolic blood pressure
	Diastolic blood pressure
	Heart failure score
*Laboratory*	BNP/NT-BNP
	Creatinine
	Sodium
	Potassium
	Blood urea nitrogen
	Haemoglobin
*Drug treatment*	ACE inhibitors
	Angiotensin receptor blockers
	Beta-blockers
	Mineralocorticoid receptor antagonists
	Loop diuretic
	Thiazide diuretics
	Vasodilator
	Other potassium sparing diuretic
	Aspirin
	Other anti-platelet agent
	Oral anticoagulant
	Digoxin
	Amiodarone
	Other anti-arrhythmic
	Calcium-channel blocker
	Statin
*Quality of life*	If collected (derived scores if available)
**Individual participant**	**Clinical outcomes (data required for all deaths, hospital admissions or cardiovascular events)**
	Date of death
	Cause of death
	Date of hospital admission/cardiovascular event
	Date of hospital discharge
	Details of reason for admission/cardiovascular event (for example heart failure, non fatal myocardial infarction, non-fatal stroke, new atrial fibrillation, fitting of pacemaker/CRT device/implantable cardioverter defibrillator)
*Lost to follow-up?*	Date of last follow-up

ACE, angiotensin-converting enzyme; CABG, coronary artery bypass grafting CRT, cardiac resynchronization therapy; BNP, B-type natriuretic peptide; NT-BNP, N-terminal pro-B-type natriuretic peptide; NYHA, New York Heart Association; PCI, percutaneous coronary intervention.

Raw data can be transferred by a variety of secure methods (courier, secure email, or secure electronic transfer) depending on the preference of the authors’ institutions. All raw data will be stored on a secure server. Raw datasets will be saved in their original formats and then converted to a common format by renaming and labeling the variables for each study in a consistent manner. We will develop a framework for mapping and classifying sufficiently similar variables; this will be discussed and agreed within the Collaborative Group, and carefully documented.

#### Data checking

We will carry out various checks on the data (see examples below), and discuss and clarify discrepancies with study authors. For each variable, we will calculate the proportion of missing observations and compare this with data in the original publication. We will carry out range checks for all included variables to ensure that all values are reasonable, and tabulate categorical variables and check against the values tabulated in the original publication. Datasets will be combined to form a new master dataset, including a variable indicating the original study.

### Data analysis

#### IPD meta-analysis

We will use standard meta-analysis methods incorporating all available IPD. All analyses (other than for adverse effects) will be performed on an intention-to-treat basis. The primary outcome will be time to all-cause mortality, defined as the time from randomization to death from any cause, which will be analyzed by survival methods. HRs will be estimated using Cox regression models within each trial. The estimated log HRs will be combined across studies using standard fixed-effect meta-analysis and random-effects meta-analysis, along with prediction intervals to represent heterogeneity. Appropriate regression models will be used to estimate ORs for adverse effects (taking a per-protocol approach). Standardized mean differences will be computed for quality of life if different scales are used in different trials.

Subgroup effects will be estimated by estimating treatment × covariate interaction terms within studies, and combining these across studies in the same way [12]. Covariates defining subgroups will be: age, gender, type of HF, severity of HF, baseline BNP levels, NYHA class, LVEF, BP, BMI, AF, diabetes, cardiomyopathy, and risk score for mortality. Further analyses will investigate study-level variables such as BNP-guided intervention characteristics (comparisons to be specified when study-level data have been extracted) using meta-regression. Forest plots will be produced for overall effects and for interaction effects. Interaction effects will be interpreted by applying them to meta-analyses of the participants in a reference subgroup (for example, when looking at the effect in males versus females, the meta-analytic interaction coefficient will be added to a meta-analytic estimate of the HR for females only to obtain an estimate of the HR for males). As above, we intend to present random-effects estimates, along with prediction intervals to represent heterogeneity.

#### Inclusion of aggregate data

For trials that do not provide IPD, we will undertake additional analyses in which we combine IPD when they are available with aggregate data when they are not. We will seek estimates of HRs from reports of studies not providing IPD [19] and combine these with estimated HRs derived from the IPD. If HRs are unavailable for a substantial proportion of participants, we will implement a combined analysis of the IPD and aggregate data, using all-cause mortality (rather than time to all-cause mortality) and a Bayesian hierarchical model [20]. Such a model also allows limited investigation of patient-level effect modifiers, by separating within-study and between-study relationships [14].

#### Sensitivity analysis

We plan to conduct a sensitivity analysis by restricting the analysis to trials classified as having low risk of bias overall. We will also conduct a sensitivity analysis restricting the analysis to trials with good allocation concealment, as this has been shown to be an important source of bias in RCTs [21,22]. We may additionally implement one-stage models for IPD as sensitivity analyses [23,24].

#### Checking for publication and data availability bias

We will follow the recommendations set out by Ahmed *et al*. [25] to examine the likelihood of publication bias and data availability bias. We will examine funnel plots to investigate association between study size and effect size (which could be due to publication bias), with and without studies lacking IPD. We will describe study-level and patient-level characteristics of included studies. We will report the meta-analysis of IPD from trials that have supplied IPD, and a meta-analysis that combines the individual participant data with the aggregate data from the trials lacking individual participant data (if we do not obtain IPD from all the eligible trials).

### Further development of Statistical Analysis Plan

The analyses described above are the main analyses we propose to conduct. Modifications and/or additional analyses may be indicated as the project progresses. We will write a more detailed statistical analysis between receipt of the data and the full data analysis, in which we will specify how covariates will be modeled (for example whether quantitative patient-level characteristics such as age are treated as continuous or categorical).

### How the IPD collaboration will work

We have set up a Management Group, including members from the Bristol Clinical Trials and Evaluation Unit (see http://cteu.bris.ac.uk/), a UKCRN-registered trials unit at the University of Bristol, and a Professor of Evidence Synthesis at the University of Bristol. The Management Group will be responsible for contacting and liaising with study authors, data collection, checking and analyses, interpretation of findings, and preparing manuscripts for publication.

The IPD meta-analysis Collaborative Group will include all members of the Management Group, clinicians with expertise in HF, a health economist, and a representative from each of the included trials. New collaborators will be invited as eligible trials are completed. The Management Group will liaise between members of the Collaborative Group and organize all necessary interactions between study members. Members of the Collaborative Group will be consulted at key stages of the project, and given the opportunity to participate in decision-making regarding the statistical analyses to be included in the Statistical Analysis Plan and interpretation of the findings.

#### Publication policy

All publications resulting from the IPD meta-analysis will be in the name of the Collaborative Group, with the contribution of members being described at the end of the paper. Manuscripts will be prepared by the Management Group and circulated to all members of the Collaborative Group for comment prior to submission for peer review. All collaborators will be expected to consult with, and collate comments from, colleagues from the trials they represent.

### Confidentiality, data storage, and handling

The anonymized dataset supplied by each collaborator and the final IPD dataset will be used only for the purposes stated in this protocol and analyses set out in the analysis plan. Any additional analyses will require the approval of every member of the Collaborative Group. All datasets will be stored in password-protected files on a secure computer accessible only by members of the Management Group, and archived in accordance with patient data archiving procedures required by the UK National Health Service (NHS). The datasets will not be shared with anyone outside the Collaborative Group without the express permission of each collaborator.

#### Ethical issues

The IPD meta-analysis is exempt from ethics approval because we will be collecting and synthesizing data from previous clinical trials in which informed consent has already been obtained by the trial investigators, and our meta-analysis will be addressing very similar questions to the research question for which the data were collected (and to which patients gave consent). Moreover, we will request contributors only to submit pseudoanonymized datasets (that is, the key linking study number to identifiable patient data will be retained by the contributor and not shared with the project team). Each main trial author will be allowed to specify any additional restrictions on data usage or storage (beyond those stated above) that they may wish to impose.

#### Dissemination plans

Findings from both meta-analyses will be reported in line with the Preferred Reporting Items for Systematic Reviews and Meta-Analyses (PRISMA) [21]. We will also adhere to additional reporting guidelines recommended for IPD meta-analysis [16].

## Discussion

This IPD meta-analysis will update an existing IPD meta analysis of 8 RCTs [13]. It will allow us to estimate the effect of BNP-guided therapy on clinical outcomes, how patient characteristics modify treatment benefit, and identify relevant subgroups of patients who are likely to benefit most from BNP-guided therapy.

### Registration

The protocol for the IPD meta-analysis has been registered with PROSPERO, the international prospective register of systematic reviews, (PROSPERO 2013: CRD42013005335).

## Abbreviations

ACE: Angiotensin-converting enzyme; BNP: B-type natriuretic peptide; CI: Confidence interval; HR: Hazard ratio; HF: Heart failure; IPD: Individual participant data; NHS: National Health Service; NT-BNP: N-terminal pro-B-type natriuretic peptide; NYHA: New York Heart Association; OR: odds ratio; PRISMA: Preferred Reporting Items for Systematic Reviews and Meta-Analyses; RCT: Randomized controlled trial; RR: Relative risk.

## Competing interests

The authors declare that they have no competing interests.

## Authors’ contributions

MP: conception and design, manuscript writing and final approval of the manuscript; JH: methodological and statistical advice and final approval of the manuscript; CR: methodological and statistical advice, and final approval of the manuscript; LD: critical revision of the manuscript and final approval of the manuscript; WH: health economics perspective and final approval of the manuscript; MD: clinical advice, critical revision of the manuscript and final approval of the manuscript; AN: clinical advice, critical revision of the manuscript, and final approval of the manuscript; TM: clinical advice, critical revision of the manuscript, andfinal approval of the manuscript; BR: conception and design, critical revision of the manuscript, and final approval of the manuscript. All authors have read and approved the final manuscript.

## Funding

This study is part of a project funded by the National Institute of Health Research (NIHR) Health Technology Assessment (HTA 11/102/03). The views and opinions expressed are those of the authors, and do not necessarily reflect those of the HTA program, NIHR, the UK NHS, or the Department of Health.

## Supplementary Material

Additional file 1 Search strategy: MEDLINE (Ovid) 1950 to present. Click here for file
