# Supplementary material for: Protocol for a systematic review and individual participant data meta-analysis of B-type natriuretic peptide-guided therapy for heart failure
Source: Syst Rev. 2014 May 2;3:41. doi: 10.1186/2046-4053-3-41 (PMC4113204; doi:10.1186/2046-4053-3-41)
Supplement: Additional file 1 — Search strategy: MEDLINE (Ovid) 1950 to present. [file 2046-4053-3-41-S1.doc]

**Additional file 1. Search strategy**

**Medline (Ovid) 1950 to present**

--------------------------------------------------------------------------------

Database: Medline 1950 to present

Search Strategy:

--------------------------------------------------------------------------------

1 (BNP adj5 (guide$ or monitor$ or target$ or predict$)).tw. (875)

2 (proBNP adj5 (guide or monitor$ or target$ or predict$)).tw. (585)

3 (NTproBNP adj5 (guide$ or monitor$ or target$ or predict$)).tw. (30)

4 ((natriuretic peptide or natriuretic propeptide) adj5 (guide$ or monitor$ or target$ or predict$)).tw. (770)

5 ((NTproBNP or Natriuretic Peptide or natriuretic propeptide or BNP or proBNP) adj5 (retest$ or serial or series)).tw. (177)

6 ((NTproBNP or Natriuretic Peptide or natriuretic propeptide or BNP or proBNP) adj5 (manag$ or tailor$ or treat$ or therap$ or strateg$)).tw. (1034)

7 or/1-6 (2734)

8 exp Heart Failure/ (88614)

9 heart failure.tw. (103737)

10 cardiac failure.tw. (9777)

11 HF.tw. (19875)

12 CHF.tw. (10524)

13 or/8-12 (152446)

14 Natriuretic Peptide, Brain/ (9880)

15 Monitoring, Physiologic/ (45222)

16 "Predictive Value of Tests"/ (145475)

17 "Health Status Indicators"/ (21035)

18 or/15-17 (209672)

19 14 and 18 (1512)

20 (BNP adj5 (guide$ or monitor$ or target$ or predict$)).tw. (875)

21 (proBNP adj5 (guide or monitor$ or target$ or predict$)).tw. (585)

22 (NTproBNP adj5 (guide$ or monitor$ or target$ or predict$)).tw. (30)

23 ((natriuretic peptide or natriuretic propeptide) adj5 (guide$ or monitor$ or target$ or predict$)).tw. (770)

24 ((NTproBNP or Natriuretic Peptide or natriuretic propeptide or BNP or proBNP) adj5 (retest$ or serial or series)).tw. (177)

25 ((NTproBNP or Natriuretic Peptide or natriuretic propeptide or BNP or proBNP) adj5 (manag$ or tailor$ or therap$ or strateg$)).tw. (540)

26 or/20-25 (2324)

27 19 or 26 (3187)

28 13 and 27 (1767)

29 randomized controlled trial.pt. (390995)

30 controlled clinical trial.pt. (90070)

31 randomized.ab. (288395)

32 placebo.ab. (157299)

33 drug therapy.fs. (1772029)

34 randomly.ab. (200079)

35 trial.ab. (303857)

36 groups.ab. (1280166)

37 or/29-36 (3308511)

38 exp animals/ not humans/ (4066609)

39 37 not 38 (2817704)

40 28 and 39 (622)

41 ("2012$" or "2013$" or "2014$").ed. (1700243)

42 40 and 41 (105)

**Embase (Ovid) <1980 to 2012 Week 26>**

--------------------------------------------------------------------------------

1 exp heart failure/ (240304)

2 heart failure.tw. (125430)

3 cardiac failure.tw. (11574)

4 CHF.tw. (13935)

5 HF.tw. (26271)

6 or/1-5 (284966)

7 brain natriuretic peptide/ (13290)

8 monitoring/ (68421)

9 predictive value/ (18283)

10 "disease course"/ (253029)

11 "symptom"/ (82438)

12 disease course/ (253029)

13 "pathophysiology"/ (552261)

14 patient monitoring/ (57907)

15 biological monitoring/ (11401)

16 hemodynamic monitoring/ (11474)

17 8 or 9 or 10 or 11 or 12 or 13 or 14 or 15 or 16 (1015916)

18 7 and 17 (2351)

19 (BNP adj5 (guide$ or monitor$ or target$ or predict$)).tw. (1252)

20 (proBNP adj5 (guide or monitor$ or target$ or predict$)).tw. (813)

21 (NTproBNP adj5 (guide$ or monitor$ or target$ or predict$)).tw. (111)

22 ((natriuretic peptide or natriuretic propeptide) adj5 (guide$ or monitor$ or target$ or predict$)).tw. (894)

23 ((NTproBNP or Natriuretic Peptide or natriuretic propeptide or BNP or proBNP) adj5 (retest$ or serial or series)).tw. (236)

24 ((NTproBNP or Natriuretic Peptide or natriuretic propeptide or BNP or proBNP) adj5 (manag$ or tailor$ or treat$ or therap$ or strateg$)).tw. (1373)

25 or/19-24 (3676)

26 18 or 25 (5641)

27 6 and 26 (3599)

28 random$.tw. (734627)

29 factorial$.tw. (18994)

30 (crossover$ or cross-over$).tw. (61441)

31 placebo$.tw. (175748)

32 (doubl$ adj blind$).tw. (128507)

33 (singl$ adj blind$).tw. (12267)

34 assign$.tw. (204642)

35 allocat$.tw. (68787)

36 volunteer$.tw. (157058)

37 Crossover Procedure/ (34246)

38 Double-blind Procedure/ (109462)

39 Randomized Controlled Trial/ (324293)

40 Single-blind Procedure/ (16047)

41 or/28-40 (1210587)

42 (animal/ or nonhuman/) not human/ (4452630)

43 41 not 42 (1063367)

44 27 and 43 (461)

45 limit 44 to embase (395)

**The Cochrane Library**

--------------------------------------------------------------------------------

#1 MeSH descriptor Heart Failure explode all trees

#2 "heart failure"

#3 "cardiac failure"

#4 CHF

#5 HF

#6 (#1 OR #2 OR #2 OR #4 OR #5)

#7 MeSH descriptor Natriuretic Peptide, Brain, this term only

#8 (BNP near/5 (guide* or monitor* or target* or predict*))

#9 (NTproBNP near/5 (guide* or monitor* or target* or predict*))

#10 (("natriuretic peptide") near/5 (guide* or monitor* or target* or predict*))

#11 ((NTproBNP or "Natriuretic Peptide" or "natriuretic propeptide" or BNP or proBNP) near/5 (retest* or serial or series))

#12 ("natriuretic propeptide" near/5 (guide* or monitor* or target* or predict*))

#13 (NTproBNP or "Natriuretic Peptide" or "natriuretic propeptide" or BNP or proBNP):ti

#14 (NTproBNP or "Natriuretic Peptide" or "natriuretic propeptide" or BNP or proBNP) near/5 (manag* or tailor* or therap* or strateg*)

#15 (proBNP near/5 (guide* or monitor* or target* or predict*))

#16 (#7 OR #8 OR #9 OR #10 OR #11 OR #12 OR #13 OR #14 OR #15)

#17 (#6 AND #16)

**ISI Web of Science (Citations Index and Conference Proceedings)**

--------------------------------------------------------------------------------

# 18 #17 AND #16

# 17 TS=(random* or trial or placebo* or groups (double same blind*) or (single same blind*))

# 16 #15 AND #1

# 15 #14 OR #13 OR #12 OR #11 OR #10 OR #9 OR #8 OR #7 OR #6 OR #5 OR #4 OR #3 OR #2

# 14 TS=((NTproBNP or "Natriuretic Peptide" or "natriuretic propeptide" or BNP or proBNP) NEAR (strateg*))

# 13 TS=((NTproBNP or "Natriuretic Peptide" or "natriuretic propeptide" or BNP or proBNP) NEAR (therap*))

# 12 TS=((NTproBNP or "Natriuretic Peptide" or "natriuretic propeptide" or BNP or proBNP) NEAR (tailor*))

# 11 TS=((NTproBNP or "Natriuretic Peptide" or "natriuretic propeptide" or BNP or proBNP) NEAR (manag*))

# 10 TS=((NTproBNP or "Natriuretic Peptide" or "natriuretic propeptide" or BNP or proBNP) NEAR series)

# 9 TS=((NTproBNP or "Natriuretic Peptide" or "natriuretic propeptide" or BNP or proBNP) NEAR serial*)

# 8 TS=((NTproBNP or "Natriuretic Peptide" or "natriuretic propeptide" or BNP or proBNP) NEAR retest*)

# 7 TS=("natriuretic propeptide" NEAR (guide* or monitor* or target* or predict*))

# 6 TS=("natriuretic peptide" NEAR (guide* or monitor* or target* or predict*))

# 5 TS=(NTproBNP NEAR (guide* or monitor* or target* or predict*))

# 4 TS=(proBNP NEAR (guide or monitor* or target* or predict*))

# 3 TS=(BNP NEAR (guide* or monitor* or target* or predict*))

# 2 TS=("natriuretic peptide" NEAR target*) or TS=("natriuretic propeptide" NEAR target*)

# 1 TS=("heart failure" or "cardiac failure" or CHF or HF)
